# The Citric Acid Cycle Modulates Neurologic Health and Is a Therapeutic Target of Dietary and Genetic Modification in Metabolic Disease

**DOI:** 10.3390/genes17020192

**Published:** 2026-02-04

**Authors:** Keri J. Fogle, Sarah K. Lindley, Sidney L. Satterfield, Beakal A. Amsalu, Joseph R. Figura, Samantha L. Eicher, Luke A. Scherz, Michael J. Palladino

**Affiliations:** 1 Department of Pharmacology & Chemical Biology, University of Pittsburgh School of Medicine, Pittsburgh, PA 15260, USA; kjf38@pitt.edu (K.J.F.); sarah.lindley2@pennmedicine.upenn.edu (S.K.L.); sls378@pitt.edu (S.L.S.); jof149@pitt.edu (J.R.F.); lscherz@som.geisinger.edu (L.A.S.); 2 Pittsburgh Institute for Neurodegenerative Diseases, University of Pittsburgh School of Medicine, Pittsburgh, PA 15260, USA

**Keywords:** citric acid cycle, genetics, mitochondrial encephalomyopathy, anaplerosis, ketogenic diet, Leigh Syndrome, circadian rhythms, nucleoside diphosphate kinase

## Abstract

**Background/Objectives:** Primary metabolic diseases including mitochondrial encephalomyopathies (ME), glycolytic enzymopathies, and disorders of lipid and amino acid metabolism can manifest with severe neurological and neuromuscular symptoms. Conversely, it is increasingly appreciated that primary neurodegenerative diseases can have metabolic etiology and pathophysiology. Pharmacological treatments have limited benefit for these classes of diseases, but dietary therapy is increasingly recognized as a tool for bolstering metabolic processes that can ameliorate neurological symptoms. The ketogenic diet is the best-established example, having long been used as a therapy for epilepsy. Replenishing metabolic intermediates (anaplerosis) especially substrates of the citric acid cycle (CAC) is currently being explored, with ongoing clinical trials of simple metabolic intermediates such as oxaloacetate or NAD+ to treat neurodegenerative diseases. We have shown ketogenic and anaplerotic therapies to be effective in a *Drosophila* model of ME; however, the full therapeutic potential and role of the CAC in neuronal health is still not well understood. **Methods:** Here, we have used genetic, behavioral, and dietary approaches to elucidate critical links between the CAC and neurological function. **Results:** We have found that stimulating the CAC can improve and sustain neurological health in the face of severe metabolic disease, and that its functions include a previously unrecognized role in maintaining normal circadian rhythms, whose disruption is often an early indicator or complicating factor in neurological and neurodegenerative disease. We investigated the hypothesis that the production of GTP by the CAC may be an important mechanistic contributor to the role of the CAC in neurological health and disease, and may underlie its therapeutic potential. **Conclusions:** Overall, our findings expand our understanding of the role of the CAC in neurological health and disease, support its development as a therapeutic target, and provide a foundation for further studies investigating the intersection between neurological disease and metabolic function.

## 1. Introduction

Neurological and neurodegenerative diseases are increasingly recognized to share etiology, pathophysiology, and potential therapeutic approaches with metabolic diseases [1,2,3,4], which can originate in any of the three major branches of cellular respiration—glycolysis, the citric acid cycle (CAC), or mitochondrial oxidative phosphorylation (OXPHOS). For example, glucose hypometabolism is an early biomarker of Alzheimer’s Disease (AD) [5,6], the most common form of dementia affecting over 50 million people worldwide. Additionally, downregulation of glycolytic, CAC, and OXPHOS enzymes have been reported in critical brain areas with AD [7,8]. Mitochondria in the AD brain can have altered bioenergetics, excess ROS production, and abnormal structure, mitophagy, and biogenesis [3,7,9]. Overwhelming evidence suggests that pathogenesis of Parkinson’s Disease (PD), too, involves significant mitochondrial disruption [10,11,12]. Mitochondrial complex I toxins such as MPTP, rotenone, and paraquat induce loss of dopaminergic neurons in the substantia nigra, a key hallmark of PD [13]. Additionally, mutations in both mtDNA and nuclear genomes have been linked to PD pathogenesis, and the α-ketoglutarate dehydrogenase complex has been implicated in some cases of spontaneous PD [14].

Furthermore, the class of neurological disorders collectively referred to as epilepsy are worsened by oxidative stress caused by genetic mutation or mitochondrial dysfunction [15,16,17]. Abnormal mitochondrial fission is frequently observed in seizure disorders, including temporal lobe epilepsy [18]. The robust link between metabolism and hyperexcitability that leads to seizures is also apparent in the long-established success of the ketogenic diet (KD). Dramatically reducing glycolysis in favor of fatty acid oxidation and ketogenesis with a KD is a metabolic switch that attenuates refractory seizures of several etiologies [19,20,21,22].

Conversely, primary metabolic diseases often present with neurological and neuromuscular symptoms, owing in part to the high energetic demand of the nervous system. Symptoms of mitochondrial encephalomyopathies (ME) and metabolic enzymopathies affecting glycolysis, the CAC, and other metabolic pathways include intellectual disability, developmental delay or regression, cognitive or sensory impairment, movement or mobility issues, disordered sleep, and seizures [23,24,25,26,27,28,29,30]. These effects may arise from bioenergetic defect, buildup of harmful metabolites such as methylglyoxal or lactic acid, and/or the generation of damaging reactive oxygen species.

Leigh Syndrome (LS) is a devastating and incurable disease of mitochondrial energy production that typically manifests in pediatric patients with symptoms including seizures, ataxia, dystonia, vision problems, and lactic acidosis; it is currently incurable and carries a uniformly poor prognosis [31,32]. This mitochondrial encephalomyopathy and the related disorders Neuropathy Ataxia Retinitis Pigmentosa (NARP) and Familial Bilateral Striatal Necrosis (FBSN) are caused by mutations in the mitochondrially encoded gene encoding ATP6, the proton-passing subunit of Complex V (ATP synthase) [33,34]. The *Drosophila* model of these disorders, *ATP6^1^*, has a missense mutation resulting in a glycine to glutamate amino acid substitution at position 116 that precludes ATP production from OXPHOS. The *ATP6^1^* strain has high mutant heteroplasmy, is stable and maternally inherited, and faithfully models human ME, exhibiting seizures, muscle degeneration, locomotor impairment, circadian rhythm defects, and a significantly reduced lifespan [35,36,37,38].

We have recently shown that the ketogenic diet dramatically improves the seizure severity of *ATP6^1^*; [39] we also found that one of the major downstream consequences of the KD, anaplerosis (bolstering of the CAC), is sufficient to ameliorate seizures via supplementation of a standard carbohydrate-based diet with the highly anaplerotic synthetic lipid triheptanoin [40]. Here, these promising therapeutic insights led us to further evaluate anaplerotic treatment for seizures and overall health in mitochondrial disease, as well as to determine the involvement of this metabolic hub in the perturbed circadian rhythms in *ATP6^1^*. We also used a genetic approach to investigate GTP/ATP production by the CAC as a mechanism underlying anaplerotic benefit. Overall, we show that proper function of the CAC is critical to neurological health in both healthy and diseased animals, and that anaplerotic therapies may succeed in part by increasing ATP availability.

## 2. Materials and Methods

### 2.1. Drosophila Genetics

Strains, matings, and experimental flies were maintained in standard media comprising agar, corn meal, dextrose, sucrose, dry yeast, and molasses, with propionic acid, phosphoric acid, and Tegosept added as fungicides. Flies are maintained in a light- and temperature-controlled room under a 12:12LD schedule. In all reported genotypes, “*ATP6^1^*” or “*ATP6* [1]” refers to the mitochondrially encoded gene mutation in *ATP6* and is denoted first, separated from the nuclear chromosomes by standard semi-colon notation. The *ATP6^1^* strain used in all experiments was a mitochondrial recombinant generated by the O’Farrell lab and described previously [41,42]. The control strain is *ATP6^+^; w^1118^*.

*Knockdown*, *idh *(null), *elavGAL4*, and *UAS-RNAi* lines were obtained from Bloomington FlyStocks (Bloomington, IN, USA). The *UAS-NDPK* overexpression line was generated by FlyORF (University of Zurich). *UAS/GAL4* transgenic progenies were generated using standard genetic methods with *ATP6[1]*; w*; *elavGAL4* stable line virgin females mated to males with the *UAS* construct of interest. Double mutant *ATP6[1]; w; +; TPI^sgk/sgk^* flies (AKA *sugarkill*) were generated using a standard multi-step mating scheme with chromosome III balancers to facilitate homozygous *TPI^sgk^*, and were maintained under standard laboratory conditions as a stable line.

### 2.2. Specialty Media

Administration of the ketogenic diet was performed as described previously [39,40]. Briefly, a standard vial was filled with an agar plug for hydration while the ketogenic media paste was provided in a 1 cm plastic cup (generated by clipping a 1 mL disposable filter pipette tip) embedded in the agar plug for nutrition. The ketogenic paste comprised a 3:1 ratio by mass of coconut oil:carbohydrates (sucrose, dextrose, 20% of the yeast, and 80% of the corn meal content of standard media).

Lipids and supplements were obtained from Sigma (St. Louis, MO, USA), except for triheptanoin which was obtained from BOC Sciences (Shirley, NY, USA). Lipids (pentadecanoic acid, lauric acid, and triheptanoin) were mixed directly into melted standard media at the indicated concentration (*w*:*v*). Intermediates/metabolites were first dissolved in an aqueous stock solution and then added to the indicated concentration in large batches of melted standard media which were then aliquoted into empty standard vials for treatment. Flies were maintained on specialty media starting three days before seizure or circadian behavioral testing and from eclosion for lifespan assays.

### 2.3. Mechanical Stress Seizure Induction

Experiments testing stress sensitivity or bang sensitivity (BS) to assay stress-induced seizures were conducted as originally described by Ganetzky and Wu [43] with some modifications that have been described in detail previously [36]. Briefly, flies are placed in empty plastic, cotton-plugged vials in groups of 5–8, then vortexed at maximum speed for 20 s. Bang-sensitive flies exhibit paralysis, convulsions, or a combination of the two, while wild type flies recover immediately following the stimulus. *ATP6^1^* and *TPI^sgk^* flies exhibit progressively longer periods of paralysis and convulsions; due to this phenotype, their seizure severity is measured by the parameter time to recovery (TTR), which measures how long it takes each fly to resume purposeful movements after paralysis and convulsion. Flies are generally tested every three days starting at the onset of disease (early), mid-life (intermediate), and after severe disease has set in (advanced). The exact timing of the phenotype progression depends on disease model and temperature (room temp 21–22 °C or an incubator at 25 °C). Flies were provided with fresh standard or supplemented media every three days.

### 2.4. Lifespan Assays

Flies were collected on day of eclosion, and cohorts of 20–30 males and females were placed on either standard or supplemented media immediately. Every two days each vial was checked and the number of deaths recorded; the remaining flies were then provided with a fresh vial. Incidental losses (escape, crushing, drowning in condensation, etc.) were censored and not included in the death rate. These were infrequent and <~5–10% of the total animals.

### 2.5. Circadian Assays

Sleep and 24 h activity rhythm experiments were performed using standard TriKinetics (Waltham, MA, USA) *Drosophila* activity monitors which generate activity profiles by recording the breaking of an individual infrared beam due to animal movement within the apparatus. Each monitor holds up to 32 flies, whose activity is recorded in one-minute bins as described previously [37]. Male flies of indicated genotypes were collected simultaneously at eclosion and maintained in the 12:12LD 25-degree C incubator for entrainment. *W^1118^* controls of *ATP6^1^* flies and transgenic lines derived from them were loaded at ages 14–16 days and their behavior analyzed from days ~15–19. *ATP6[1];;;TP^sgk^* flies, because of their earlier onset disease, were loaded into monitors at age ~7–8 days and analyzed from days ~8–12. Flies were entrained before loading and immediately placed into DD incubators for data collection.

Daily/nightly activity, actograms, circadian period, and rhythm strength were calculated/constructed using ShinyR-DAM [44]. Multiple trials of the same genotype under the same conditions were combined and averaged in Excel using the downloaded .csv data from ShinyR-DAM. N values represent the number of individual flies except in percent rhythmicity where n represents number of trials, with each trial corresponding to one monitor loaded with 32 individuals of the indicated genotype (total number of flies in these trials is reported in rhythm strength and period length descriptions).

### 2.6. Data Analyses

Data are presented as bars with error bars representing mean ± standard error, with all individual data points contributing to the set overlaid. Data were tested for normality using the Shapiro–Wilk test and many behavioral data sets failed normality testing. Therefore, two-way comparisons were performed by Mann–Whitney tests with two-tailed *p*-values and multiple value comparisons were performed by Kruskal–Wallis tests followed by Dunn’s multiple comparisons tests. Lifespan data were analyzed using a logrank (Mantel–Cox) test followed by the Holm–Sidak multiple comparisons test. Data were compiled and organized in Excel and then, using Prism, were analyzed and figures were generated. * = *p* < 0.05; ** = *p* < 0.01; *** = *p* < 0.001; **** = *p* < 0.0001. 

## 3. Results

### 3.1. The CAC Is a Promising Therapeutic Target for ME

The KD has been known since antiquity to reduce epileptic seizures and is still used clinically, especially in cases of drug-refractory epilepsies. We have shown that it dramatically improves the seizures originating from mitochondrial disease in *ATP6^1^*, perhaps a somewhat surprising result given that its metabolic hallmarks—increased fatty acid oxidation and ketogenesis—still ultimately provide energy to neurons via mitochondrial ATP production, which is perturbed in *ATP6^1^* due to a loss of OXPHOS. We thus explored other mechanistic possibilities for KD success and discovered that direct supplementation of ketone bodies also improves seizure phenotypes [39,40]; this could be proceeding via ion channel modulation, histone modification leading to gene expression changes, post-translational modification of enzymes, or other mechanisms [20,22,45,46,47,48,49].

**Figure 1:**
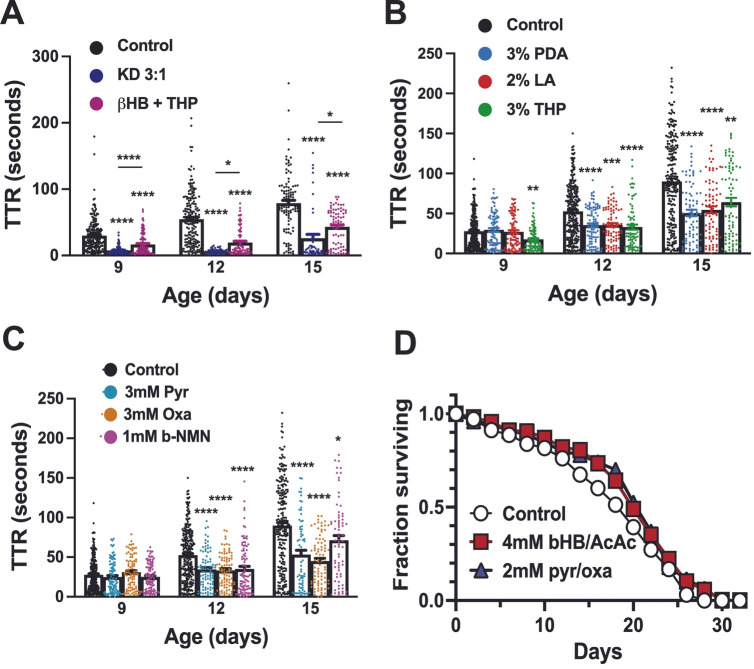
Anaplerotic supplementation of carbohydrate-based diet improves seizure phenotype and lifespan. (**A**) At all tested time points (ages 9, 12, and 15 days), control media-fed flies’ seizure TTR (white bars) is significantly improved (*p* < 0.0001) by the KD (dark blue points) or by supplementation with ketone bodies (b-hydroxybutyrate 1 mM) plus 3% THP (magenta points). These two treatments, while both effective, are different from each other (*p* < 0.0001 for Day 9; *p* = 0.0260 for Day 12; *p* = 0.0225 for Day 15). Day 9: Control 29.8 ± 1.7, n = 202; b + T 16.6 ± 1.4, n = 117; KD 7.5 ± 0.6, n = 109. Day 12: Control 54.6 ± 3.0 n = 181; b + T 18.7 ± 2.1 n = 85; KD 6.7 ± 0.5 n = 46. Day 15: Control 78.8 ± 4.4 n = 107; b + T 43.0 ± 3.1 n = 78; KD 26.0 ± 6.1 n = 47. (**B**) Lipid enrichment of carbohydrate-based media using pentadecanoic acid (PDA, light blue points) and lauric acid (LA, red points) confer a significant seizure amelioration benefit that is comparable to that of THP (green points) during intermediate and late disease. *p* < 0.0001 for PDA and THP on Day 12 and PDA and LA on Day 15, vs. control. *p* = 0.0005 for LA on Day 12 and 0.0019 for THP on Day 15. Additionally, THP improves TTR significantly over control (*p* = 0.0031) on Day 9 (early disease). Day 9: Control 27.81 ± 1.3 n = 269; 3% PDA 29.4 ± 2.25 n = 92; 2% LA 26.91 ± 2.2 n = 89; 3% THP 17.63 ± 1.48 n = 94. Day 12: Control 52.57 ± 2.0 n = 272; 3% PDA 35.385 ± 2.4 n = 90; 2% LA 35.7 ± 2.4 n = 80; 3% THP 32.98 ± 3.2 n = 77. Day 15: Control 90.0 ± 3.6 n = 233; 3% PDA 50.59 ± 4.0 n = 80; 2% LA 54.57 ± 4.2 n = 75; 3% THP 65.16 ± 5.3 n = 76. (**C**) Supplementation of carbohydrate-based media with anaplerotic metabolites pyruvate (Pyr) and oxaloacetate (Oxa) significantly improves seizure severity (*p* < 0.0001) at intermediate (Day 12) and advanced (Day 15) disease time points. NADH precursor b-nicotinamide mononucleotide (b-NMN) also significantly improves seizures vs. control on Day 12 (*p* < 0.0001) and Day 15 (*p* = 0.049). Day 9: Control 27.81 ± 1.3 n = 269; 3 mM Pyr 24.82 ± 2.0 n = 96; 3 mM Oxa 30.84 ± 2.2 n = 88; 1 mM NMN 25.18 ± 1.69 n = 101. Day 12: Control 52.57 ± 2.0 n = 272; 3 mM Pyr 34.42 ± 2.5 n = 89; 3 mM Oxa 33.31 ± 3.2 n = 89; 1 mM NMN 35.08 ± 3.3 n = 94. Day 15: Control 90.0 ± 3.6 n = 233; 3 mM Pyr 53.160 ± 5.5 n = 67; 3 mM Oxa 45.2 ± 3.3 n = 83; 1 mM NMN 71.29 ± 5.9 n = 72. (**D**) Supplementation with ketone bodies (*p* < 0.0001) and anaplerotic intermediates (*p* < 0.01) improved lifespan significantly. At 14 days, 67.4% survived on control media compared to 80.5% with ketone body supplements and 78% with anaplerotic intermediates. Median survival was 20 days for all groups. * = *p* < 0.05; ** = *p* < 0.01; *** = *p* < 0.001; **** = *p* < 0.0001.

We found that another major downstream consequence of the KD, the elevated availability of intermediates to the CAC (anaplerosis), provides a strong therapeutic benefit in seizure amelioration. The synthetic lipid triheptanoin (THP), which is easily metabolized into both acetyl-CoA and succinyl-CoA, improves seizures significantly when supplemented into a standard carbohydrate-based diet [40,50,51,52]. The combination therapy with the ketone body b-hydroxybutyrate and 3% THP dramatically reduces seizure time to recovery (TTR) at all time points (Figure 1A).

The ability of supplemental treatments to function in the presence of normal carbohydrate levels is promising, as, in practice, the KD is difficult to adhere to and may have adverse cardiovascular and gastrointestinal side effects, especially in pediatric patients or those with other severe disease complications. THP is effective but difficult to attain and expensive. Thus, we asked whether anaplerotic substrates that are more readily available from dietary and other accessible sources could provide some of the same benefits.

Pentadecanoic acid (PDA, C15:0), an odd-chain highly anaplerotic fatty acid found in dietary sources such as whole milk and cheese, beef, lamb, lard, fish, and certain seeds and oils, and lauric acid (LA), a ketogenic medium chain triglyceride that is the major component of coconut oil, were tested for seizure amelioration potential alongside THP. Both of these common dietary components significantly improved seizure time to recovery (TTR) at intermediate and advanced time points (Figure 1B). The results of supplementation with 3 mM pyruvate and 3 mM oxaloacetate, an immediate precursor and essential intermediate, respectively, of the CAC demonstrate that direct consumption of these metabolites can also provide therapeutic benefit in ME seizures, significantly improving TTR (*p* < 0.0001) at intermediate and advanced time points (Figure 1C). Additionally, supplementation of a standard diet with the direct NAD+ precursor b-nicotinamide mononucleotide (b-NMN) significantly improved seizure phenotype at intermediate (*p* < 0.0001) and advanced disease (*p* = 0.0494) time points vs. control media (Figure 1C).

We also performed lifespan assays on *ATP6^1^* flies on standard media, standard media supplemented with ketone bodies (2 mM each b-hydroxybutyrate and acetoacetate), and standard media supplemented with oxaloacetate and pyruvate (1 mM each). Both of these interventions significantly improved lifespan (*p* < 0.0001 for ketone bodies and *p* < 0.01 for intermediates), suggesting these supplementation strategies improve not just seizure/hyperexcitability but healthspan in general in ME (Figure 1D).

### 3.2. Flies Lacking Both OXPHOS and Glycolytic ATP Are Viable and Benefit from Ketogenic and Anaplerotic Treatments

These findings demonstrate that treatment focusing on augmenting the CAC can imbue neurological benefit in mitochondrial disease and emphasizes the importance of the CAC in cellular function in the face of metabolic dysfunction. To test this idea further, we created mutant flies that contained both the *ATP6^1^* mutation and the *TPI^sgk^* mutation in the essential glycolytic enzyme triosephosphate isomerase (TPI), which catalyzes the interconversion of dihydroxide acetone phosphate (DHAP) to glyceraldehye-3-phosphate (G3P) [53,54,55]. The TPI product G3P can be further processed in the glycolytic pathway producing ATP, reducing equivalents, and pyruvate, while DHAP cannot. Thus, flies with both of these mutations (*ATP6^1^;;TPI^sgk^*) lack both OXPHOS-generated ATP and are severely impaired in their ability to generate ATP through glycolysis (although importantly, pyruvate can still be produced and processed to enter the CAC). Two independent genetic lines with both of these mutations were generated and their phenotypes tested in parallel (strain A and B).

**Figure 2:**
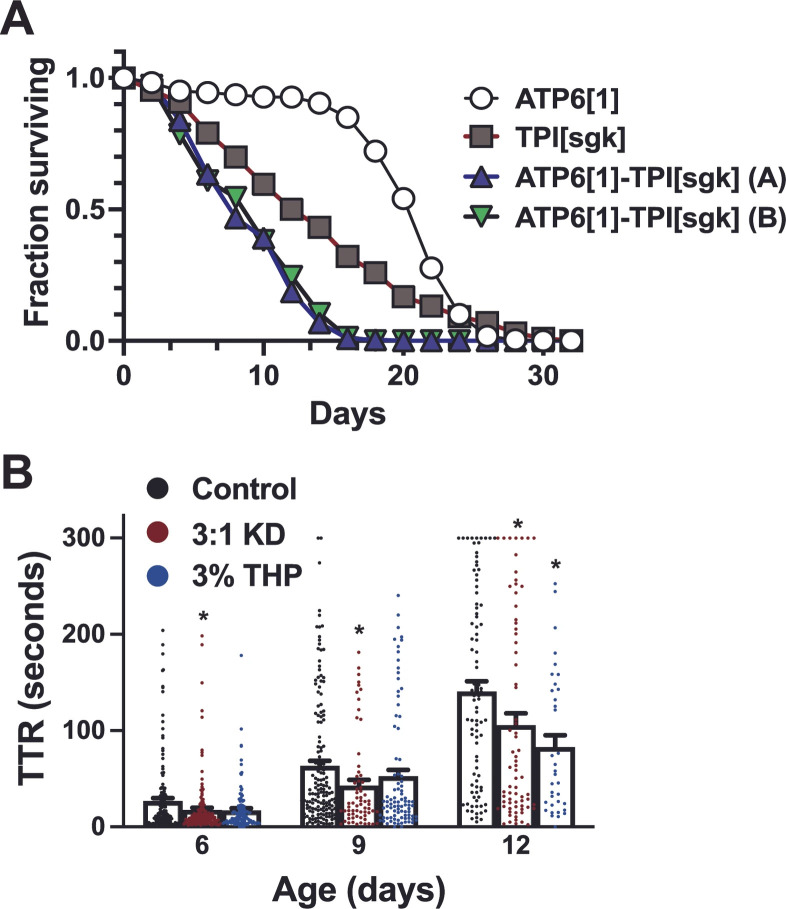
Double *ATP6[1]–TPI[sgk]* mutants survive and respond to dietary therapy. (**A**) Flies carrying only the ATP6[1] mitochondrial mutation (white circles) reach median survivorship at Day 22 and drop below 5% at Day 26 while those with only TPI[sgk] (gray boxes) reach median at Day 14 and 5% at Day 28. In contrast, animals with both mutations (green and blue triangles) reach median survival on Day 8 (*p* < 0.0001) and have maximal lifespan of 18–20. Survival of ATP6[1] vs. TPIsgk, ATP6[1] vs. double mutant lines, and TPIsgk vs. double mutant lines is significantly different (*p* < 0.0001). Double mutant line A vs. B, *p* < 0.05. (**B**) ATP6[1]–TPI[sgk] double mutant flies experience severe, early-onset seizures, with a time to recovery of 27.05 ± 3.0 (n = 167) at Day 6. KD improves TTR at Day 6, 17.6 ± 2.4 (n = 151, *p* = 0.033 vs. control); 3% THP, 17.15 ± 2.1 (n = 121, *p* = 0.19 vs. control). At Day 9, KD treatment is significantly lower than control (*p* = 0.033) but THP does not reach significance (*p* = 0.072). Control: 63.5 ± 5.3 n = 158; KD: 43.0 ± 6.1 n = 65; THP: 52.585 ± 5.7 n = 90. At Day 12, the KD improves TTR significantly (*p* = 0.046) as does anaplerotic treatment with THP (*p* = 0.022). Control: 140.64 ± 10.7 n = 90; KD: 105.8 ± 12.3 n = 72; THP: 82.9 ± 12.3 n = 36. TTR data is compiled from the two identical genetic lines shown in A and B, which are not different from each other under any of the displayed conditions. * = *p* < 0.05.

*ATP6^1^;;;TPI^sgk/sgk^* flies are viable at room temperature conditions (20–22 °C) and fertile, reliably producing enough offspring to maintain the next generation. As *TPI^sgk/sgk^* is a well-described temperature-sensitive mutant that shows mild neuromuscular dysfunction at room temperature but displays increasingly severe phenotypes including seizures and reduced coordination with elevations in temperature [53], we examined the lifespans of animals containing this mutation under incubation at 25 °C. This assay reveals that at elevated temperature, their disease state is greater in severity than either *ATP6^1^* or *TPI^sgk/sgk^* alone (Figure 2A), reaching 50% survival by 8–10 days and achieving a maximum lifespan of ~two weeks. In comparison, *TPI^sgk/sgk^* flies exhibit 50% survival at 15 days and a maximum lifespan of 28 days, while *ATP6^1^* flies reach these milestones at Days 21 and 26, respectively (*p* < 0.0001 for all comparisons between distinct genotypes and *p* < 0.05 between the two double mutant lines). Median lifespan was 22 days for *ATP6^1^*, 14 days for *TPI^sgk/sgk^*, and 8 days for each of the double mutant lines.

*ATP6^1^;;;TPI^sgk^* flies also exhibit earlier onset and more rapid progression of seizures than *ATP6^1^* alone (compare to Figure 1), with substantially lengthened TTR within the first week of life, and minutes-long paralysis upon mechanical stimulation by age 12 days (Figure 2B), even when assessed at room temperature (22 °C). Despite these severe symptoms, treatment with ketogenic and anaplerotic diets (10% THP in regular media) can successfully improve TTR at some time points. (Day 6; *p* = 0.0334 for KD and *p* = 0.1958 for THP). Recovery times at intermediate disease (Day 9) reach significance for the KD (*p* = 0.0330) but not THP (*p* = 0.0729). However, at Day 12, both the KD (*p* = 0.0461) and THP (*p* = 0.0221) improve seizures significantly vs. control, suggesting anaplerotic treatment is as beneficial as the ketogenic treatment in advanced compound metabolic disease.

**Figure 3:**
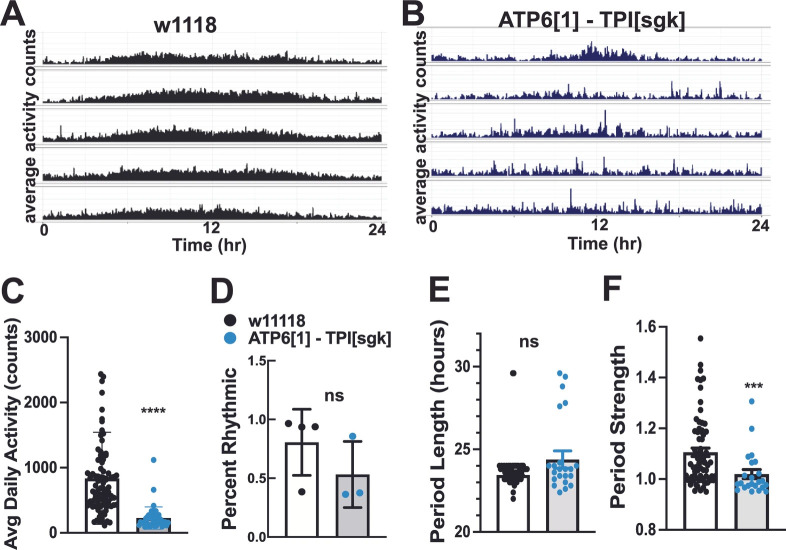
Double *ATP6[1]–TPI[sgk]* mutants have reduced activity and perturbed circadian rhythms. (**A**) Example average actogram for genetic background control line w1118. Each row represents the activity pattern for one day under constant darkness conditions (DD) at 25 °C. (**B**) Example average actogram for *ATP6[1]–TPI[sgk]* double mutant flies. (**C**) Average daily activity is drastically reduced in double mutant flies (gray bar, light blue symbols 228.18 ± 26.2 n = 44) vs. control (white bars, black symbols, 837.17 ± 75.3 n = 89; *p* < 0.00001). (**D**) Percent of flies that reach rhythmicity threshold for control (80.6 ± 15 n = 4 trials) vs. double mutants (53.2 ± 19.9, n = 3 trials, *p* = 0.1143). (**E**) Free-run period length vs. control (23.44 ± 0.2 n = 70) is lengthened for double mutants (24.38 ± 0.54 n = 23) but both are within normal limits (*p* = 0.3727). (**F**) Rhythmicity robustness is reduced in double mutants (1.02 ± 0.02, n = 23) vs. controls (1.10 ± 0.02, n = 70, *p* = 0.0004). *** = *p* < 0.001; **** = *p* < 0.0001.

The overall neurological and neuromuscular health of *Drosophila* can be further assessed using activity monitors (see Methods). When placed in monitors which record minute-by-minute activity (Trikinetics DAM system), the double mutant flies exhibit low average daily activity (228.18 ± 26.2, n = 44) vs. control (837.17 ± 75.3, n = 89; *p* < 0.00001), as displayed in the average actograms vs. control (Figure 3A–C). When placed in constant darkness, *ATP6^1^;;;TPI^sgk^* flies also display a profound inability to maintain circadian rhythms (Figure 3D), with only 53.2% (n = 3 trials) of flies displaying measurable rhythmicity compared to 80.6% of age-matched (~7–14 days) controls. Robustness of the circadian period of those flies that were rhythmic is also significantly reduced in the *ATP6^1^;;;TPI^sgk^* double mutants (1.02 ± 0.02, n = 23) vs. controls (1.10 ± 0.02, n = 70, *p* = 0.0004).

### 3.3. Genetic Interference with the CAC in the Brain Produces Defects in Circadian Rhythms

Despite their severe disease, *ATP6^1^;;;TPI^sgk^* double metabolic mutants are viable and fertile and can be maintained under standard laboratory conditions, and their neurologic function can be improved with dietary conditions that promote ketogenesis and anaplerosis. Since the only genetically wildtype branch of cellular respiration present in these flies is the CAC, this supports the idea that this metabolic hub plays a major role in maintaining the health of metabolic and bioenergetic compromised animals. Additionally, it is well-established that CAC mutation can lead to neurological defects such as seizures [56,57,58]. With these facts in mind, we reasoned that the CAC may also have previously unrecognized roles in neurological health; since both mitochondrial encephalomyopathy flies [37] and double metabolic mutant flies exhibit compromised circadian rhythms, we examined CAC mutant flies for similar defects in rhythmicity.

**Figure 4:**
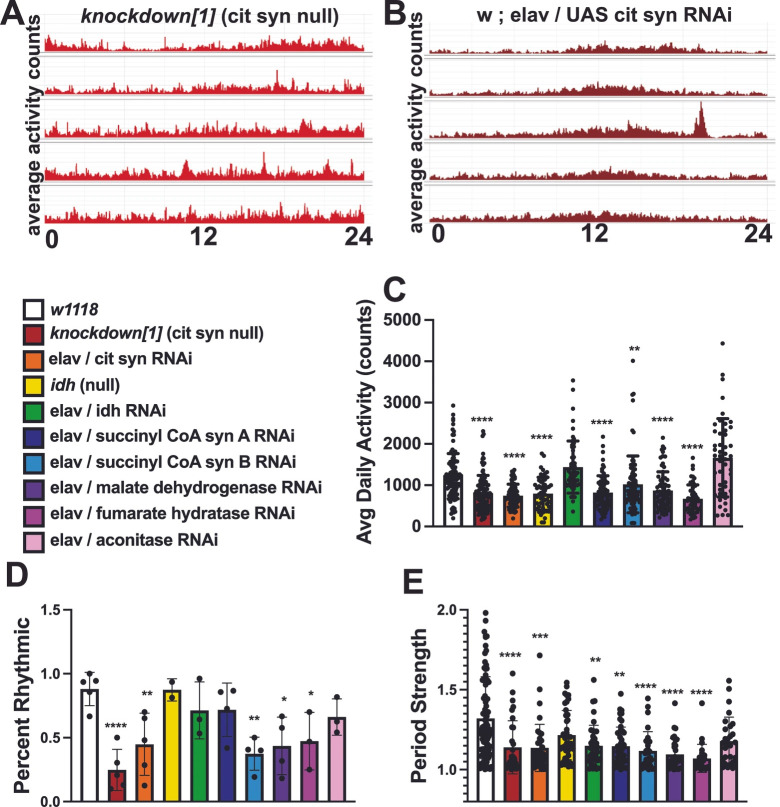
Genetic disruption of CAC enzymes impair activity levels and circadian rhythms. (**A**) Example average actograms for *knockdown[1]*, citrate synthase amorphic allele. (**B**) Example actogram for pan-neuronally driven RNAi against kdn/citrate synthase show lack of robust rhythmicity. (**C**) Genetic disruption of citrate synthase (*knockdown[1]*, red bar, 803.6 ± 42, n = 109) and isocitrate dehydrogenase (idh null, yellow bar, 792 ± 56, n = 51) significantly reduces daily activity (*p* < 0.0001) compared to control (white bars, 1216 ± 54, n = 103). Pan-neuronally expressed RNAi against kdn/citrate synthase, two subunits of succinyl CoA synthetase, malate dehydrogenase, and fumarase also dramatically and significantly reduce daily activity levels (*p* < 0.0001 for all except succ coA b, *p* = 0.0072). (**D**) *knockdown[1]* dramatically reduces percent rhythmicity compared to control (88.1 + 6.5, n = 5 vs. 24.8 ± 8.1, n = 5, *p* < 0.0001). RNAi against kdn/citrate synthase, succinyl CoA synthetase subunit b, malate dehydrogenase, and fumarase also significantly reduce percent rhythmicity (44.8 ± 12.2, n = 5, *p* = 0.0086; 37.5 ± 7.5, n = 4, *p* = 0.0033; 43.6 ± 12.9, n = 4, *p* = 0.0112, 47.3 ± 13.7, n = 3, *p* = 0.0429). (**E**) Period robustness was significantly reduced for *knockdown[1]*, succinyl CoA synthetase b, malate dehydrogenase, fumarase (*p* < 0.0001), citrate synthase RNAi (*p* = 0.0002), idh RNAi (*p* = 0.0046), and succinyl CoA synthetase a (*p* = 0.0010). * = *p* < 0.05; ** = *p* < 0.01; *** = *p* < 0.001; **** = *p* < 0.0001.

The classical bang-sensitive allele *knockdown* (*kdn*) arises from an amorphic allele of citrate synthase [56]. We tested both males and females of this genotype and found a profound loss of circadian rhythmicity, as demonstrated by an erratic activity pattern in the actogram (Figure 4A). We also used a transgenic RNAi approach to interfere with citrate synthase expression in the brain (w*; *elavGAL4/UAS-RNAi cit syn*, see methods), and found that the rhythmicity of these flies is also impaired (Figure 4B).

To follow up on this result, we additionally tested brain-specific RNAi knockdown driven by pan-neuronal driver *elavGAL4* of other CAC enzymes in a wild type (non-ME) genetic background, including isocitrate dehydrogenase, aconitase, succinyl-CoA dehydrogenase subunits, fumarate hydratase, and malate dehydrogenase for defects in circadian rhythms. Brain-specific RNAi directed at the genes encoding CAC enzymes of otherwise healthy flies, significantly reduced circadian rhythm percentage and strength (Figure 4C–E), indicating that proper CAC functioning is critical to maintaining normal circadian rhythms and underscoring the physiological links between this metabolic cycle and neurological function.

### 3.4. Nucleoside Diphosphate Kinase (NDPK) Is Necessary for Seizure Amelioration by the Ketogenic Diet in ME

**Figure 5:**
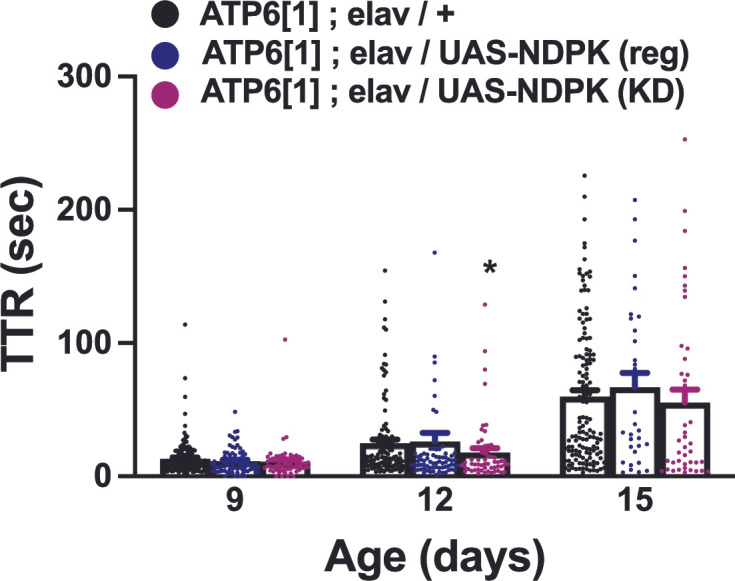
RNAi knockdown of nucleoside diphosphate kinase (NDPK) prevents the KD from seizure amelioration. There is no significant difference in seizure TTR between driver-only controls (*ATP6[1]*; elav/+, black symbols) and pan-neuronally driven NDPK RNAi in an *ATP6[1]* background (*ATP6[1]*; *elav/UAS-NDPK RNAi*) on regular media (dark blue symbols) at all days tested or KD (magenta symbols) at Day 9 or 15. Control vs. NDPK RNAi on KD on Day 15, *p* = 0.018. Day 9: Control 13.2 ± 0.9, n = 169; NDPK reg 11.34 ± 1.1, n = 75; NDPK KD 10.89 ± 1.8, n = 59. Day 12: Control 24.95 ± 2.7, n = 119; NDPK reg 26.23 ± 6.5, n = 74; NDPK KD 17.95 ± 3.25, n = 55. Day 15: Control 59.9 ± 4.7, n = 144; NDPK reg 66.9 ± 10.9, n = 32; NDPK KD 55.3 ± 9.85, n = 43. * = *p* < 0.05.

The links between the CAC, metabolic disease, and dietary therapy are likely to be complex and multifaceted. However, we hypothesized that one likely source of interconnection in the face of extreme bioenergetic stress, such as that which occurs in an animal that lacks OXPHOS or both OXPHOS and proper glycolytic ATP production, is the ability of the CAC to produce GTP that can be utilized for cellular energy. Each turn of the CAC produces a single GTP via succinyl CoA synthetase as it converts succinyl CoA to succinate. The major enzyme that converts GTP to ATP is nucleoside diphosphate kinase (NDPK), which uses a histidine phosphorylation “ping-pong” mechanism to transfer a phosphate between ATP and another nucleoside diphosphate [59,60]. In flies, this enzyme is encoded by the gene *awd* (*abnormal wing disks*) [61]. To test the importance of this GTP/ATP interconversion to the neurological health of *ATP6^1^*, we used the pan-neuronal driver *elavGAL4* to drive the RNAi of *awd* and reduce NDPK expression and then tested seizures and their response to the KD. *awd* RNAi has no basal effect on seizure severity of *ATP6^1^* that is fed a regular diet (Figure 5). However, when these RNAi flies are treated with the KD, instead of amelioration, there is a surprising lack of change in seizure phenotype severity at the early, intermediate, and advanced time points. This finding supports the hypothesis that the synthesis of ATP via the conversion of GTP produced by the CAC is a critical component of the success of the KD’s therapeutic benefit in ME.

### 3.5. Overexpression of NDPK Improves Lifespan, Seizures, and Circadian Rhythms in ME

**Figure 6:**
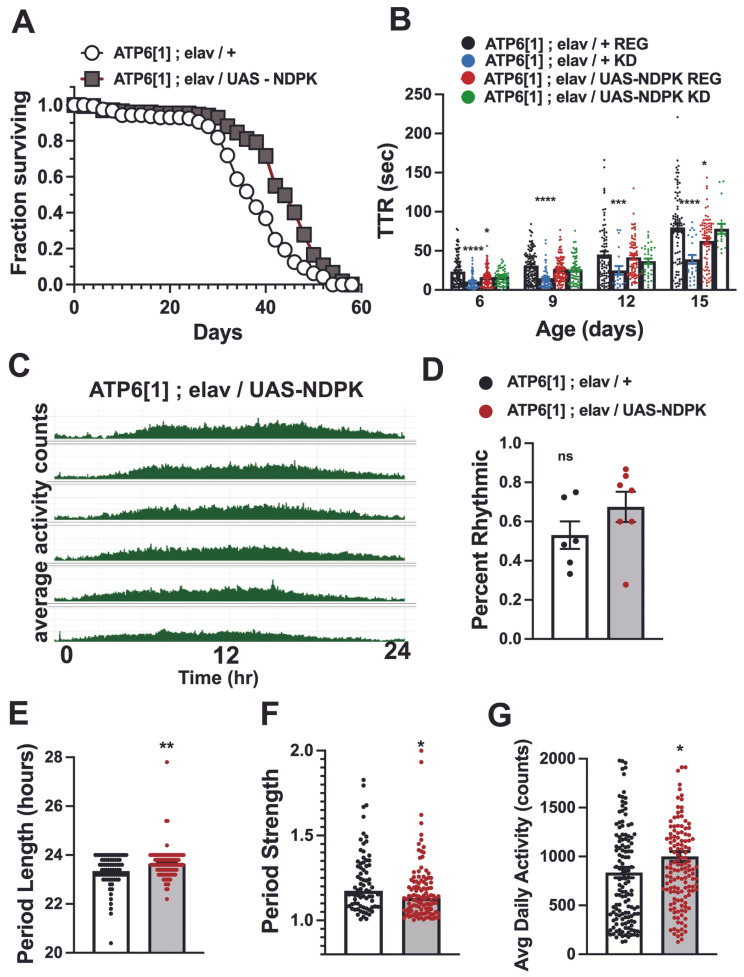
Pan-neuronal overexpression of NDPK improves lifespan, seizure phenotype, and circadian parameters. (**A**) Flies overexpressing NDPK in the brain (*ATP6[1]; elav/UAS-NDPK*, gray boxes) show increased survival at all time points including 50% (45 days vs. 37 for control, white circles, *p* < 0.0001), 33% (47 days vs. 41 for control), and 10% (53 days vs. 47 for control). (**B**) Day 6: Control 23.46 ± 1.9, n = 103; KD 10.75 ± 1.25, n = 62; UAS-NDPK reg 16.435 ± 1.24, n = 92; UAS-NDPK KD 16.5 ± 1.24, n = 59. Control vs. KD, *p* < 0.0001; Control vs. UAS-NDPK reg, *p* = 0.0375. Day 9: Control 31.2 ± 1.76, n = 117; KD 14.8 ± 1.9, n = 55; UAS-NDPK reg 26.5 ± 1.5, n = 105; UAS-NDPK KD 25.9 ± 2.1, n = 65. Control vs. KD, *p* < 0.0001. Day 12: Control 47.52 ± 4.1, n = 82; KD 24.8685 ± 5.8, n = 20; UAS-NDPK reg 41.6 ± 2.9, n = 75; UAS-NDPK KD reg 36.7 ± 3.5, n = 34; Control vs. KD, *p* = 0.0095. Day 15: Control 80.0 ± 5.1, n = 83; KD 39.1 ± 6.5, n = 23; UAS-NDPK reg 62.65 ± 3.8, n = 74; UAS-NDPK KD 78.2 ± 6.4, n = 19. Control vs. KD, *p* < 0.0001; Control vs. UAS-NDPK reg, *p* = 0.0461. (**C**) Representative average actogram for ATP6[1] overexpressing NDPK in the brain. (**D**) UAS-NDPK expression improves percent rhythmicity to 67.5% ± 8.3 (n = 7) over 53% ± 7.6 (n = 6) for driver-only controls (ATP6[1]; w; elav/+), but does not reach significance *p* = 0.1930. ns is not significant. (**E**) Period length for driver controls is 23.3 ± 0.07 (n = 78) and 23.6 ± 0.06 (n = 111, *p* = 0.0057) for UAS-NDPK overexpressing flies. (**F**) Rhythm strength for driver controls is 1.225 ± 0.02 (n = 78) and 1.175 ± 0.02 (n = 111, *p* = 0.0124) for UAS-NDPK overexpressing flies. (**G**) UAS-NDPK overexpression increases ATP6[1] average daily activity to 1000.25 ± 50.8 (n = 144) over driver controls (835.35 ± 53.4, n = 139, *p* = 0.0044). * = *p* < 0.05; ** = *p* < 0.01; *** = *p* < 0.001; **** = *p* < 0.0001.

To test this idea further, we overexpressed NDPK using a *UAS-NDPK* transgene with pan-neuronal expression throughout the brain of *ATP6^1^* animals and tested lifespan, seizure, and circadian rhythm parameters. We reasoned that if interconversion of GTP to ATP was a contributing factor in ketogenic and anaplerotic neurological benefit in ME and this might represent a rate-limiting step in the process, then genetic upregulation of the major enzyme catalyst of this reaction may lead to improvements in neurological function. In support of our hypothesis, we found that the NDPK transgenic overexpression did result in a measurable benefit, extending median survivorship by 8 days, from 37 to 45 (*p* < 0.0001). (Figure 6A).

We also tested the seizure phenotype of *ATP6^1^* overexpressing NDPK and found that it modestly but significantly improved TTR over driver control flies at Days 6 and 15 (*p* = 0.0375 and *p* = 0.0461, respectively) (Figure 6B). At Days 9 and 12, the overexpression of NDPK did not reduce seizure TTR. Intriguingly, this genetic manipulation, although it improved basal seizure phenotype, removed the ability of the flies to benefit from the KD—there was no significant further improvement in TTR in NDPK overexpressing flies fed a KD at any tested time point.

Finally, we tested the circadian rhythms of the NDPK overexpression animals, with an average actogram showing a visible daily rhythm pattern (Figure 6C). The percent of rhythmic animals was 67.5% ± 8.3 (n = 7 trials), higher than in driver-only controls at 53% ± 7.6 (n = 6 trials). The period length was also improved, at 23.6 ± 0.06 (n = 111, *p* = 0.0057), which is closer to a normal 24 h period than driver controls at 23.3 ± 0.07 (n = 78). Period robustness/strength was reduced (*p* = 0.0124), demonstrating that although rhythmicity was improved, its robustness was not. Encouragingly, overexpression of NDPK improved overall daily activity levels from 835.35 ± 53.4 (n = 139) for controls to 1000.25 ± 50.8 (n = 144, *p* = 0.0044), suggesting that the production of ATP via this pathway may contribute to neurological and neuromuscular performance.

## 4. Discussion

### 4.1. Anaplerosis as a Translational Therapy in ME

Despite its well-documented effectiveness against refractory seizures and emerging evidence of benefits to a wide range of neurological disorders, the clinical use of the ketogenic diet has significant drawbacks. Maintaining strict ketosis is difficult, labor-intensive, and may be unsafe for vulnerable groups including pediatric patients, those with cardiovascular or digestive issues, and those managing multifaceted states of disease. Therefore, finding ways to recreate the downstream effects of a KD is a critical line of therapeutic investigation.

Direct consumption of ketone bodies, often accomplished in humans by ingestion of ketone esters [62], has proven promising, and in our mitochondrial encephalomyopathy model reduces seizures and extends lifespan. Here we have shown that ketogenic and anaplerotic lipids, pentadecanoic acid and lauric acid, that are found in readily available dietary sources can improve neurological hyperexcitation when consumed as a significant proportion of the diet. Importantly, this benefit is seen even when carbohydrates comprise a substantial portion of the caloric content. Translationally, this suggests that increased consumption of certain fats can improve health parameters even when ketosis is not achieved.

Pyruvate, oxaloacetate, b-nicotoinamide mononucleotide, and other CAC intermediates and precursors such as a-ketoglutarate and succinate are currently available as over-the-counter supplements. While their overall health benefits remain to be thoroughly and rigorously evaluated in the context of both healthy and diseased patients, our current results suggest that supplementation with these metabolites may provide moderate neurological benefit in a *Drosophila* model of mitochondrial encephalomyopathy. Further studies are needed to determine the potential benefits of combination therapies involving high lipid content, anaplerotic supplementation, and reduction in carbohydrate intake, but it is noteworthy that oxaloacetate, a-ketoglutarate, and NADH supplements have been shown to benefit AD models and patients [63,64,65,66,67] and are being investigated for safety and effectiveness in clinical trials [68,69,70,71]. Our results suggest clinical trials of these key citric acid cycle metabolites with patients of metabolic disease should include anaplerotic lipids such as PDA and LA. Other downstream effects of the ketogenic diet, such as ATP-sensitive K channel modulation, histone deacetylase inhibition, and antioxidant properties can also be investigated as individual or combination pharmacological, supplemental, or dietary strategies.

### 4.2. The Citric Acid Cycle as a Critical Factor in Maintaining Healthy Circadian Rhythms

Metabolic disorders involving enzymes, intermediates, or metabolic pathways that intersect the CAC are known to have neurological consequences [29,56,57,58]. In *Drosophila*, a citrate synthase null allele (*knockdown*, *kdn*) is among a handful of classical “bang sensitive” genotypes which are susceptible to mechanically induced seizures due to sensory overstimulation. We have previously shown that in the *ATP6^1^* model of mitochondrial encephalomyopathy, interfering with the CAC can worsen seizures and prevent the full benefit of ketogenic and anaplerotic therapies [40]. Circadian rhythms are a complex neurological function highly susceptible to metabolic disruption, including in mitochondrial encephalomyopathy [28,37,72,73,74,75,76]. For these reasons, we tested whether CAC dysfunction alone was sufficient to interfere with the ability of *Drosophila* to rely upon their physiological clock. This internal clock is based on a transcriptional–translational loop that cycles approximately every 24 h in a subset of circadian control neurons and responds to environmental cues such as light and temperature; in animals with a healthy functioning clock, normal rhythmicity is present, robust and stable at ~24 h in length even in the absence of external stimuli that inform the animal about time of day.

*Kdn* flies exhibited severely compromised circadian rhythms, and transgenic RNAi interference with citrate synthase in the brain mimicked these results, thus demonstrating a clear link between citrate synthase function and circadian rhythms. Additionally, knocking down other CAC enzymes—malate dehydrogenase, fumarate hydratase, and succinyl-CoA synthase—also interfered with the ability of otherwise healthy flies to maintain a robust circadian rhythm. These results can be followed up in the future to investigate the possibility of specificity in the causative relationships between specific CAC enzyme function and circadian rhythm maintenance.

Further studies will be needed to uncover the mechanistic basis of this specificity, but possible links between circadian rhythms and the CAC abound. For example, the light-sensitive regulatory protein cryptochrome requires a FAD/H cofactor that utilizes a redox reaction to detect and respond to light [77]. In mammals, the DNA binding activity of TTFL proteins BMAL/CLOCK is modified by the redox environment via the ratio of NAD/NADH and NADP/NADPH [78]. Additionally, the treatment of flies with hydrogen peroxide or the genetic overexpression of the superoxide dismutase enzyme that produces it suppresses daily locomotor rhythms [79], suggesting the pathogenic nature of ROS imbalance in circadian rhythms [80,81].

### 4.3. The CAC as a Critical Source of ATP in Metabolic Disease

We show that the CAC, as the only genetically unaltered branch of cellular respiration in *ATP6^1^*;;;*TPI^sgk^* double mutant animals, is capable of supporting a shortened but reproductively successful lifespan. This indicates that this metabolic hub may play an outsized role in energy production in the face of catastrophic metabolic failure due to lack of OXPHOS and drastically reduced glycolytic ATP production. Notably, these animals can also still benefit from ketogenic and anaplerotic dietary therapy; this stands in contrast to animals with compromised CAC function, which do not display neurological improvement in response to the KD [40]. Together, these results suggest that in this glycolytic–mitochondrial encephalomyopathy double model that does not perform OXPHOS and already has reduced glycolytic flux, the ketogenic diet is working by affecting some other process within or downstream of the CAC that improves neurological health. Importantly, the KD is anaplerotic by more than one mechanism, highlighting its potential mechanistic relevance. The KD’s use of dietary fats as the primary source of energy yields acetyl-CoA via fatty acid oxidation, but it also produces the ketone body acetoacetate in large concentrations. This metabolite can be converted to acetoacetyl-CoA from succinyl-CoA, which drives the CAC forward and enhances GTP production. Our results abolishing the KD benefit by knocking down NDPK expression with RNAi indicate that this could be a major mechanism by which the KD is working in mitochondrial encephalomyopathy.

Conversely, our results with NDPK overexpression demonstrate that CAC ability to be a direct source of cellular energy via the generation of GTP and its subsequent conversion to ATP is a key aspect of its function and a promising therapeutic target: genetic enhancement of NDPK availability improves multiple ME disease parameters, including extending lifespan, reducing seizure severity, and improving circadian rhythm parameters. This mechanism could underlie both ketogenic and anaplerotic supplementation benefits.

It is also true, however, that the CAC is a complex and intricately connected metabolic hub, and thus neurological benefit that proceeds via its enhancement is likely to be similarly multifaceted. The CAC connects via its intermediates to not just glycolysis and OXPHOS, but fatty acid oxidation, ketogenesis, the pentose phosphate pathway, and the synthesis of nucleotides, amino acids, and neurotransmitters. For example, a-ketoglutarate is a precursor to glutamate and GABA, and fueling the increased synthesis of this inhibitory neurotransmitter may have an ameliorating effect on seizures. Additionally, the CAC intermediates α-ketoglutarate, succinate, and fumarate have been shown to have epigenetic effects, modulating levels of DNA methylation [30]. The electron-carrying coenzymes NADH and FADH2 produced by the CAC are also connected to neurotransmitter synthesis: NAD+ concentration affects the synthesis of serotonin from tryptophan [82], and its precursor b-nicotinamide adenine nucleotide has been reported to act directly as an inhibitory neurotransmitter [83]. Flavins, additionally, are cofactors in monamine oxidases, which inactivate monamine neurotransmitters such as serotonin, norepinephrine, and dopamine [84]. Therefore, increased concentration of FADH2 may affect excitability and related disease phenotypes. The increased production of these highly reduced intermediates may also positively affect redox state, a potentially significant mechanism in mitochondrial encephalomyopathy due to the increased presence of reactive oxygen species; the related molecule NADPH is well known to couple to reduced glutathione biosynthesis, a powerful antioxidant mechanism. Finally, ketone bodies are now recognized to post-translationally modify proteins, including enzymes of the citric acid cycle, which may alter their function [45].

## 5. Conclusions

Our results demonstrate that the CAC is a key regulator of neuronal health, with roles in maintaining fundamental properties like excitability and complex physiological and behavioral systems such as circadian rhythms. We have also shown it is a promising therapeutic target which can be enhanced by some relatively non-invasive dietary strategies, including increasing the intake of anaplerotic lipids and supplements of CAC precursors and intermediates. We show that energy production via the CAC and NDPK may play a key role in the success of the ketogenic diet and related anaplerotic treatments in mitochondrial encephalomyopathy. Given the cellular and molecular links between metabolism and neurological health, these strategies targeting the CAC may represent promising treatments not only in mitochondrial encephalomyopathy but glycolytic enzymopathies and primary neurological and neurodegenerative diseases as well.

## Data Availability

The raw data supporting the conclusions of this article will be made available by the authors on request.
